# Definitive concurrent chemoradiotherapy with paclitaxel plus carboplatin is superior to cisplatin plus 5‐fluorouracil in patients with inoperable esophageal squamous cell carcinoma using retrospective, real‐world evidence

**DOI:** 10.1002/cam4.4025

**Published:** 2021-10-27

**Authors:** Jason Chia‐Hsun Hsieh, Pin‐Chun Chiang, Tsung‐Min Hung, Yin‐Kai Chao, Yung‐Chia Kuo, Chih‐Tsung Wen, Po‐Jung Su, Meng‐Ting Peng, Huan‐Wu Chen, Hui‐Ling Liu, Hsien‐Kun Chang, Min‐Hsien Wu, Hung‐Ming Wang

**Affiliations:** ^1^ Division of Hematology‐Oncology Department of Internal Medicine New Taipei Municipal TuCheng Hospital New Taipei City Taiwan; ^2^ Division of Hematology‐Oncology Department of Internal Medicine Chang Gung Memorial Hospital at Linkou Taoyuan City Taiwan; ^3^ College of Medicine Chang Gung University Taoyuan Taiwan; ^4^ Department of Radiation Oncology Chang Gung Memorial Hospital at Linkou Taoyuan City Taiwan; ^5^ Division of Thoracic Surgery Department of Surgery Chang Gung Memorial Hospital at Linkou Taoyuan City Taiwan; ^6^ Division of Thoracic Surgery Department of Surgery New Taipei Municipal TuCheng Hospital New Taipei City Taiwan; ^7^ Department of Emergency and Critical Care Radiology Chang Gung Memorial Hospital at Linkou Taoyuan City Taiwan; ^8^ Department of Emergency and Critical Care Radiology New Taipei Municipal TuCheng Hospital New Taipei City Taiwan; ^9^ Case Manager Department of Nursing Cancer Center Chang Gung Memorial Hospital at Linkou Taoyuan Taiwan

**Keywords:** 5‐fluorouracil, carboplatin, cisplatin, definitive chemoradiotherapy, esophageal squamous cell carcinoma, paclitaxel

## Abstract

**Background:**

The optimal definitive chemotherapy regimen during concurrent chemoradiotherapy (CRT) for patients with advanced esophageal squamous cell carcinoma (ESCC) remains unclear because of conflicting evidence. This study aimed to compare the effectiveness of taxane‐based chemotherapy with that of conventional cisplatin plus 5‐fluorouracil (PF) as the chemotherapy regimen in definitive CRT for ESCC.

**Patients and Methods:**

This retrospective study included patients with ESCC who received paclitaxel plus carboplatin (PC) or PF during definitive CRT between May 2012 and February 2015 in a medical center in Taiwan. Survival outcomes were compared after adjustment for risk factors.

**Results:**

Overall, 229 patients were evaluated. Patients in the PC group had an objective response rate of 71.1% compared with the 51.4% of the PF group (*p* = 0.016). The PC group showed a significantly longer progression‐free survival (PFS, *p *= 0.002) and overall survival (OS, *p* = 0.019) than the PF group. Salvage surgery also helped prolong both the PFS and OS (*p* < 0001). Sex (male vs. female, HR, 1.831; 95% CI, 1.016–3.303), clinical stage (HR, 1.282; 95% CI, 1.069–1.537), accumulative radiation dose (≥41.4 Gy vs. <41.4 Gy; HR, 0.640; 95% CI, 0.413–0.993), salvage surgery (yes vs. no, HR: 0.412, 95% CI: 0.298–0.570), and regimen (PF vs. PC; HR, 1.514; 95% CI, 1.109–2.067) were independent prognostic factors for cancer mortality.

**Conclusion:**

Compared with the PF regimen, the PC regimen for definitive CRT yielded significantly increased response rates and longer survival times; therefore, the PC regimen may be preferable for chemotherapy for definitive CRT in patients with advanced ESCC.

## Graphical abstract

In advanced esophageal squamous cell carcinoma patients, the PC (paclitaxel plus carboplatin) regimen for definitive chemoradiotherapy yields significantly longer survival compared with the PF (cisplatin plus 5‐fluorouracil) regimen.

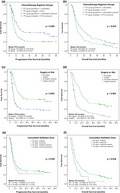

## 1 INTRODUCTION

Esophageal cancer (EC) is one of the 10 most common cancers in men worldwide.^1^, ^2^ It is highly common in Taiwan, with a crude incidence of 21.87 male cases per million and 1.73 female cases per million.^3^ Histologically, EC can be classified into esophageal adenocarcinoma (EAC) and esophageal squamous cell carcinoma (ESCC). EAC is more prevalent in North America and Europe with gastroesophageal reflux disease and obesity being the main risk factors. Meanwhile, ESCC is the more predominant EC in Asia, Africa, and South America. It is also predominant in African Americans in North America. Tobacco use and alcohol consumption are the main risk factors and esophageal squamous dysplasia is the precursor lesion.^4^ Surgery is the primary curative modality for EC, but inoperable patients are treated primarily with definitive concurrent CRT (dCRT).^5^

Chemotherapy and CRT are used as neoadjuvant therapies to improve long‐term survival.^6^, ^7^, ^8^, ^9^, ^10^ Among the chemotherapy regimens in CRT, cisplatin plus 5‐fluorouracil (PF regimen) remains the most common choice (Category 1) [9]. However, long PF infusions are associated with toxicity and intolerance, prompting the development of other regimens, in recent years. The National Comprehensive Cancer Network guideline also recommends paclitaxel and carboplatin (PC) for dCRT based on the findings of the Chemoradiotherapy for Oesophageal Cancer Followed by Surgery Study (CROSS) in 2012 that demonstrated not only a better 5‐year overall survival (OS) but also lower toxicity of PC as the chemotherapy regimen in CRT.^11^, ^12^, ^13^, ^14^

Local recurrence and distant metastasis are common after primary treatment. However, prospective trials evaluating the efficacy of different CRT regimens are lacking, and thus, the optimal regimen for dCRT or nCRT is yet to be established. Therefore, this study aimed to evaluate and compare the efficacies of PC and PF as chemotherapy regimens in CRT for EC.

## 2 MATERIALS AND METHODS

### 2.1 Study design and patients

This retrospective study was approved by the institutional review board of Chang Gung Memorial Hospital (Approval number 201701030B0).

We evaluated the patients with ESCC who were administered PC or PF during CRT between May 2012 and February 2015 at Chang Gung Memorial Hospital, Linkou, Taiwan. The inclusion criteria were: (i) age ≥20 years; (ii) Eastern Cooperative Oncology Group (ECOG) performance status 0–2; (iii) histologically confirmed ESCC; (iv) locally advanced, inoperable, newly diagnosed disease; (v) adequate liver and renal functions for dCRT; and (iv) had received chemotherapy or radiotherapy for at least 4 weeks. All patients received standard examinations for staging at diagnosis and were restaged according to the 8th edition of the American Joint Cancer Committee (AJCC) guidelines. The patients were divided into two groups, the PF and PC groups, according to their chemotherapy regimen.

### 2.2 Chemoradiotherapy protocol

After the medical oncologists discussed the differences between the regimens with the patients and their families, a clinical shared decision‐making model was used to select the regimen to be followed.

For chemotherapy, the PF regimen involved cisplatin at a dose of 60–75 mg/m^2^ on day 1 and 5‐fluorouracil (5‐FU) at a dose of 800–1000 mg/m^2^ on days 1 to 4 every 4 weeks and the PC regimen involved paclitaxel at a dose of 50 mg/m^2^ every week for six weeks. Carboplatin was delivered simultaneously at a dose calculated from an area under the curve of 2 mg/ml/min every week for 6 weeks. All the patients were premedicated intravenously with dexamethasone, cimetidine, diphenhydramine, and granisetron according to the standard protocol of the institution. All adverse effects were documented according to the National Cancer Institute's Common Terminology Criteria for Adverse Events, version 3.0.10.

Radiotherapy was performed with a total external‐beam radiation dose of 50.4–59.4 Gy in 28–30 fractions at 1.8 Gy per fraction. Radiotherapy commenced on the first day of the first chemotherapy cycle and was performed in five fractions per week. In this study, we defined the cumulative RT dose of more than 4.14 Gy as the completion threshold of CCRT.

The radiotherapy technique was based on the institution's guidelines.

### 2.3 Surgery after definitive chemoradiation

All the patients received dCRT. Surgery was performed for those who had cancer down‐staging. In these patients, surgery was performed within 4 to 6 weeks after completion of the CRT. All the pathological characteristics were documented.

### 2.4 Follow‐up

Patients who underwent surgery were followed up every 3 months in the first year, every 6 months in the second year, and then annually thereafter until 5 years. Late toxic effects, disease recurrence, and death were reviewed using data from the medical charts. Recurrence was evaluated at the time of the first recurrence. Follow‐up diagnostic investigations were performed only when recurrence was suspected. Among patients who underwent dCRT alone, those with good responses were commonly followed up with only close surveillance.

### 2.5 Statistical analysis

All the data, including the staging according to the AJCC guidelines, were revised and updated until February 2020, which guaranteed a follow‐up duration of at least 2 years. All the patients intended for dCCRT were enrolled for analysis. Age, sex, histology, clinical stage, locations of the primary tumor, radiation dose, and the number of salvage surgeries were analyzed using a simple descriptive method, while the Chi‐square or Fisher exact tests were used to compare the PF and PC groups. All the significant factors from the univariate analysis were analyzed in the multivariate Cox regression model. A forward LR model was used to evaluate statistical significance. Progression‐free survival (PFS) and overall survival (OS) were calculated from the date of diagnosis (with tissue evidence) to the date of disease progression/relapse and death, respectively. Survival curves were generated using the Kaplan–Meier method and compared using the log‐rank test. All the statistical analyses were performed using SPSS software, version 17.0 (SPSS Inc.). A *p*‐value of less than 0.05 was considered to be statistically significant.

## 3 RESULTS

### 3.1 Patient characteristics

**FIGURE 1:**
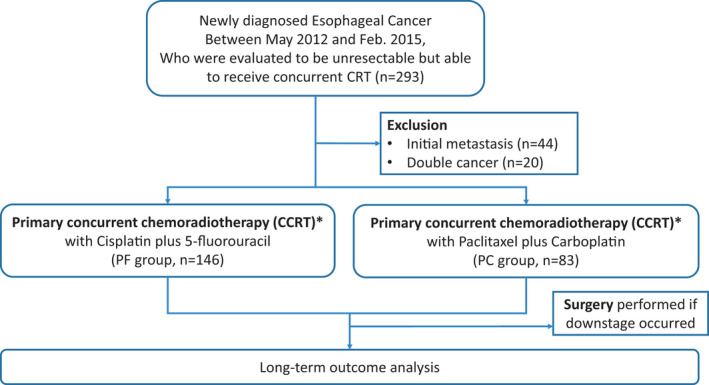
CONSORT flow diagram of patient enrollment

Of the 293 patients (including 22 patients with cervical EC) who received dCRT, 44 (15.0%) patients who had initial metastasis at diagnosis and 20 (6.8%) patients who had concurrent synchronous double cancer, were excluded. Some patients with relatively early‐stage disease (Stages IIa–IIb) also refused surgery and received dCRT mainly because of (i) their medically inoperable status or (ii) the primary location being at the cervical esophagus. Consequently, 229 patients were included in the analyses. Among them, 146 patients received the PF regimen and 83 patients received the PC regimen (Figure 1). The patients’ characteristics are shown in Table 1. The median age was 60 (range, 39–92) years. All the patients were followed until March 2020, with a median follow‐up time of 12.0 (range, 0.8–85.7) months. There were no significant differences in the clinicopathological features, including age, sex, histology, clinical stages, primary tumor locations, tumor grade, baseline ECOG performance status, cumulative radiation doses, and surgery rates, after concurrent CRT, between the PF and PC groups. In total, 78 of the 83 patients (94%) in the PC group and 133 of the 146 patients (91.1%) in the PF group were female. The majority of patients in both groups had ESCC (224 of 229, 97%). Most of the patients in both groups had stage IIIB (50.6% and 42.5% in the PC and PF groups, respectively) and stage IVA (40.9% and 49.3% in PC and PF groups, respectively) disease. The reasons for the enrolled patients being categorized as inoperable or unresectable are based primarily, on their initial stages (at least stage III, 91.5% and 91.8% in PC and PF, respectively, Table 1) and age/performance/willing to undergo surgery (8.5% and 8.2% in PC and PF, respectively, Table 1). Most tumors were located in the middle and lower part of the esophagus (PC group: 60/83 patients [72.2%]; PF group, 99/146 patients [67.8%]). Most patients had a performance status of −0–1(96.4%, 91.8%, for PC and PF groups, respectively).

**TABLE 1:** Clinicopathological patient characteristics by the group

Parameters	PC group (*n* = 83), *n* (%)	PF group (*n* = 146) *n* (%)	*p* value
Age (years), median (range)	62(42–92)	59 (39–89)	0.065
Sex			0.436
Male/female	5 (6.0%)/78 (94.0%)	13 (8.9%)/133 (91.1%)	
Histology			
SqCC	80 (96.4%)	144 (98.6%)	
Adenocarcinoma	2 (2.4%)	1 (0.7%)	
Others^a^	1 (1.2%)	1 (0.7%)	0.314
Stage (AJCC 8th edition)			
IIA‐IIB^b^	2 (2.4%)	8 (5.5%)	
IIIA	5 (6.0%)	4 (2.7%)	
IIIB	42 (50.6%)	62 (42.5%)	
IVA	34 (40.9%)	72 (49.3%)	0.467
Tumor location			
Cervical	7 (8.4%)	15 (10.3%)	
Upper third	15 (18.1%)	31 (21.2%)	
Middle third	31 (37.3%)	51 (34.9%)	
Lower third	29 (34.9%)	48 (32.9%)	0.896
Performance status (ECOG)			
0–1	80 (96.4%)	134 (91.8%)	
≥2	3 (3.6%)	12 (8.2%)	0.185
Cumulative radiation dose			
<41.4 Gy	8 (9.6%)	20 (13.7%)	
≥41.4 Gy	75 (90.4%)	126 (86.3%)	0.367
Surgery after CCRT			
No	53 (63.9%)	92 (63.0%)	
Yes	30 (36.1%)	54 (37.0%)	0.899
Grading			
GX	4 (4.8%)	21 (14.4%)	
G1	0 (0.0%)	1 (0.7%)	
G2	59 (71.1%)	101 (69.2%)	
G3	20 (24.1%)	23 (15.8%)	0.072
Response to CCRT			
CR	13 (15.7%)	9 (6.2%)	
PR	46 (55.4%)	66 (45.2%)	
SD	11 (13.3%)	24 (16.4%)	
PD	10 (12.0%)	39 (26.7%)	
Not assessed	3 (3.6%)	8 (5.5%)	0.016
Pathologic CR (*n*/total)	3/30 (10%)	6/54 (11.1%)	0.875

Abbreviations: 5‐FU, 5‐fluorouracil; CCRT, concurrent chemoradiotherapy; CR, complete remission; ECOG, Eastern Cooperative Oncology Group; G1, grade 1 well‐differentiated; G2, moderately differentiated; G3, poorly differentiated; PC, paclitaxel plus carboplatin; PD, progressive disease; PF, cisplatin+5‐fluorouracil; PR, partial response; SD, stable disease; SqCC, squamous cell carcinoma.

^a^ Other pathology includes one sarcomatoid carcinoma and one basaloid carcinoma.

^b^ Early stage (stage IIa–IIb) patients did not receive conventional surgery because of a medically inoperable status or primary location at the cervical esophagus.

With respect to treatment characteristics, there was no significant difference in the radiation dose between the two groups (accumulative doses: ≥41.4 Gy vs. <41.4 Gy, *p* = 0.367). There was also no significant difference in the proportion of patients who underwent salvage surgery between the PC and PF groups (30 (36.1%) vs. 54 (37.0%), *p* = 0.899). The reasons for the 28 dropouts in this study were (i) intolerance to CCRT (toxicity) (*n* = 18), (ii) economic issues (*n* = 3), and (iii) malnutrition or sepsis (*n* = 7).

### 3.2 Survival outcomes

**FIGURE 2:**
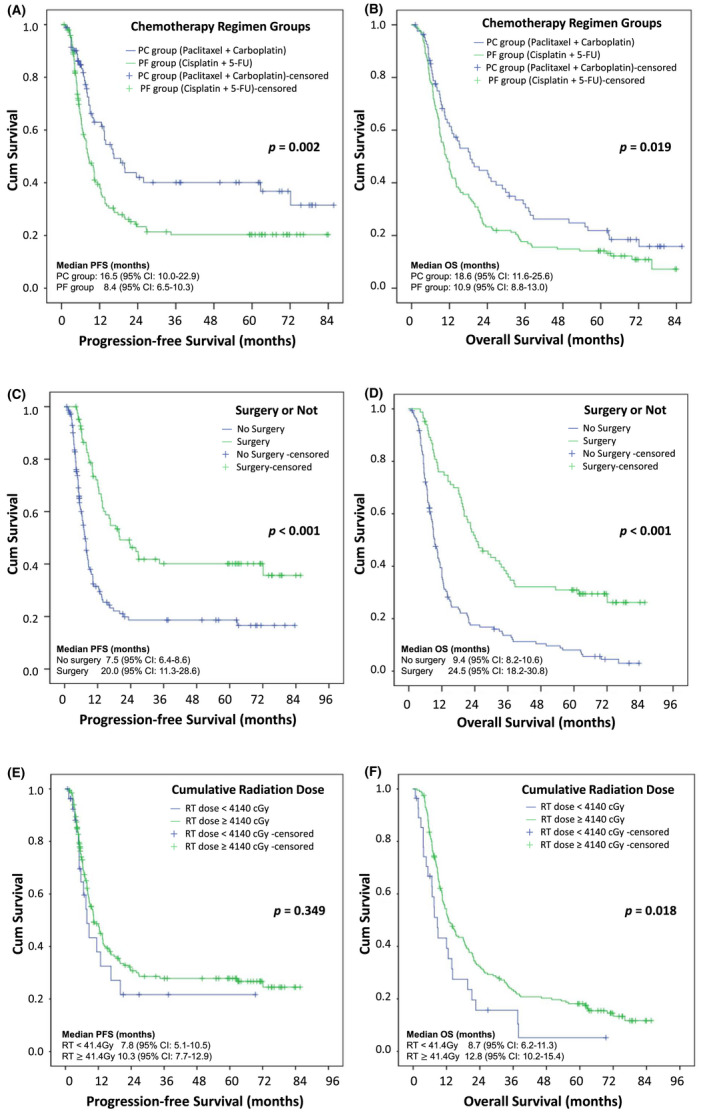
Kaplan–Meier survival curves of the two groups. The patients who received chemoradiotherapy using paclitaxel plus carboplatin (PC group) achieved significantly longer progression‐free survival (PFS, *p* = 0.002) (A) and overall survival (OS, *p* = 0.019) (B) than those who received cisplatin plus 5‐fluorouracil (PF group). Surgery after concurrent chemoradiotherapy was associated with a significantly better PFS (*p* < 0.001) (C) and OS (*p* < 0.001) (D). A cumulative radiation dose of 41.4 Gy was not related to PFS (*p* = 0.349) (E), but associated with significantly better OS (*p* = 0.018) (F)

The PC group had a significantly longer PFS (*p* = 0.002; Figure 2A, median PFS: 16.5 vs. 8.4 months) and OS (*p* = 0.019; Figure 2B, median OS: 18.6 vs. 10.9 months) than the PF group. Surgery after concurrent CRT also prolonged both the PFS (Figure 2C *p* < 0.001, median PFS: 20.0 vs. 7.5 months) and OS (Figure 2D, *p* < 0.001, median OS: 24.5 vs. 9.4 months). In addition, patients who received a higher cumulative radiation dose of ≥41.4 Gy during CRT had a longer OS (Figure 2F, *p* = 0.018, median OS: 12.8 vs. 8.7 months) than those who received less than 41.4 Gy. However, these findings were not observed with PFS (Figure 2E, *p* = 0.349, median PFS: 10.8 vs. 7.8 months).

### 3.3 Univariate and multivariate analyses of the prognostic factors

To confirm the independent prognostic factors in this cohort, we used a Cox regression model for the univariate and multivariate analyses after adjustments for all the basic factors. As shown in Table 2, the chemotherapy regimen (PF vs. PC, hazard ratio [HR], 1.840; 95% confidence interval [CI]: 1.275–2.656), AJCC stage (HR, 1.347; 95% CI, –1.096–1.657), and salvage surgery (HR, 0.417; 95% CI, 0.289–0.600) were independent prognostic factors for disease progression after concurrent dCRT. Meanwhile, sex (male vs. female; HR, 1.831; 95% CI, 1.016–3.303), AJCC stage (HR, 1.282; 95% CI, 1.069–1.537), cumulative radiation dose (≥41.4 Gy vs. <41.4 Gy; HR, 0.640; 95% CI, 0.413–0.993), salvage surgery (HR, 0.412; 95% CI: 0.298–0.570), and chemotherapy regimen (PF vs. PC; HR, 1.514; 95% CI, 1.109–2.067) were independent prognostic factors of cancer‐related mortality. Low AJCC stage at baseline, salvage surgery after definitive CRT, and chemotherapy regimen (preferably PC) can predict better survival outcomes than PF regimen in patients with locally advanced, inoperable ESCC who received dCRT.

**TABLE 2:** Univariate and multivariate analyses

Factors	Progression‐free survival				Overall survival			
	Univariate		Multivariate*		Univariate		Multivariate*	
	*p* value	HR (95% CI)	*p* value	HR (95% CI)	*p* value	HR (95% CI)	*p* value	HR (95% CI)
Age	0.433	0.993 (0.977–1.010)			0.771	0.998 (0.984–1.012)		
Sex (Male vs. female)	0.148	1.694 (0.829–3.463)			0.078	1.695 (0.943–3.049)	0.044	1.831 (1.016–3.303)
ECOG PS	0.555	1.099 (0.803–1.503)			0.117	1.246 (0.946–1.641)		
AJCC stage	<0.001	1.466 (1.187–1.810)	0.005	1.347 (1.096–1.657)	0.001	1.368 (1.139–1.642)	0.007	1.282 (1.069–1.537)
Tumor location	0.159	0.888 (0.752–1.048)			0.349	0.933 (0.806–1.079)		
Histology (SqCC vs. non‐SqCC)	0.629	0.84 5 (0.427–1.673)			0.780	1.080 (0.629–1.854)		
RT dose (≥41.4 Gy vs. <41.4 Gy)	0.352	0.781 (0.464–1.314)			0.020	0.600 (0.390–0.922)	0.047	0.640 (0.413–0.993)
Surgery (yes vs. no)	<0.001	0.397 (0.278–0.567)	<0.001	0.417 (0.289–0.600)	<0.001	0.384 (0.281–0.525)	<0.001	0.412 (0.298–0.570)
Treatment regimen (PF vs. PC)	0.003	1.749 (1.217–2.514)	0.001	1.840 (1.275–2.656)	0.020	1.436 (1.058–1.949)	0.009	1.514 (1.109–2.067)

* All the factors in the univariate analysis were examined in the multivariate model. Only factors shown to be significant in the multivariate analysis are listed in the table.

## 4 DISCUSSION

### 4.1 Novelty of the present study with comparisons

The current study reported real‐world evidence of the effectiveness of the PC regimen during dCRT in Asian patients with inoperable ESCC. We found that patients who received the PC regimen had significantly longer PFS and OS than those who received the PF regimen. In addition, the baseline AJCC stage, surgery after the CRT, and chemotherapy regimens were independent prognostic factors after adjusting for related risk factors. To the best of our knowledge, this is the largest retrospective study to report that PC had superior effectiveness to PF as the chemotherapy regimen in concurrent CRT for EC.

Table 3 summarizes the previous studies on the chemotherapy regimens for concurrent CRT, using a two‐arm comparative design. There were two main types of chemotherapy regimens in the literature, namely, the taxane‐based regimens ^11^, ^12^, ^14^, ^15^, ^16^, ^17^, ^18^, ^19^, ^20^, ^21^ (e.g., paclitaxel or docetaxel) and the platinum plus 5‐fluorouracil regimen.^22^, ^23^, ^24^, ^25^, ^26^, ^27^ Our findings were in line with those of previous studies from Japan ^20^ and China ^18^ demonstrating that patients who received concurrent CRT with a taxane–platinum regimen have more prolonged survival than those who received the PF regimen. However, it should be noted these studies used docetaxel–cisplatin, whereas we used paclitaxel–carboplatin. In contrast to our findings, Qu et al. (2017) reported that PF (*n* = 34) was superior to PC (*n* = 26) in inoperable EC.^17^ Moreover, some studies found similar outcomes between PF and PC in concurrent CRT.^14^, ^15^, ^16^, ^19^, ^21^ Fang et al. (2017) compared paclitaxel plus cisplatin with TS‐1 plus cisplatin and found that TS‐1 plus cisplatin could yield statistically better compliance in inoperable patients with EC.^15^ In their study, more than 30% (vs. the 95.6% in our cohort) of patients were inoperable. The population differences may be the reason for a non‐significant difference in the OS and PFS. Münch et al. (2017 and 2018) also reported no significant difference in the OS and relapse‐free survival between PF and PC.^16^, ^19^ However, these two studies only enrolled 25 and 31 patients who received PC, which may have been the cause of the nonsignificant impact on the OS and DFS. Horning et al. (2014) and Blom et al. (2014) both concluded that although there were no differences in the OS and DFS, PC had a comparable efficacy but lesser toxicity than PF.^14^, ^21^

**TABLE 3:** Literature review of two‐arm primary/definitive concurrent chemoradiotherapy studies in esophageal cancer

Author	Year	Country	Design	Phase	Resectability	Number of patients	Arms	Regimens and results	RT dose (Gy)	Ref.
Taxane‐platinum regimens										
Definitive chemoradiotherapy										
Tamaki et al.	2018	Japan	RS	NA	U	38+83	2	DCF>PF	60	[20]
Fang et al.	2017	China	RS	NA	U	124+79	2	P‐S1=PC	60	[15]
Münch et al.	2018	Germany	RS	NA	U	25+20	2	PF=PC	54	[16]
Qu et al.	2017	Canada	RS	NA	U	34+13+26	3	PF=CF>PC	50	[17]
Honing et al.	2014	Netherlands	RS	NA	U	47+55	2	PF=PC	50.4	[14]
Current study	2020	Taiwan	RS	NA	Inoperable	83+146	2	PC>PF	41.4–50.4	
Neoadjuvant chemoradiotherapy										
van Hagen et al.	2012	Netherlands	**PS**	III	R	178+188	2	PC +surgery > surgery alone	41.4	[12]
Xi et al.	2017	China	RS	NA	R	32+98	2	DP>PF	40	[18]
Münch et al.	2017	Germany	RS	NA	R	31+20	2	PC=PF	41.4	[19]
Haisley et al.	2017	Germany	RS	NA	R	87+55	2	PF>PC	50.4	[11]
Blom et al.	2014	Netherlands	RS	NA	R	73+92	2	PF=PC	41.4–50.4	[21]
Non‐taxane‐containing regimens in the neoadjuvant setting or in chemoradiotherapy										
Conroy et al.	2014	France	PS	III	U	134+133	2	FOLFOX=PF	50	[28]
Alderson et al.	2017	UK	PS	III	R	451+446	2	ECX=PF	no RT	[29]
Yoon et al.	2015	Korea	PS	II	R	47+50	2	ICT>no ICT	46	[22]
von Döbeln GA et al.	2019	Sweden, Norway	PS	II	R	90+91	2	nCRT=no nCRT	40	[23]
Xing et al.	2014	China	RS	N/A	U	40+35	2	CCRT=SCRT	54–60	[24]
Suh et al.	2014	Korea	RS	N/A	U	77+49	2	CCRT RT dose high>low	54	[25]
Chen et al.	2018	China	RS	N/A	U	49+41	2	CCRT=RT	56	[26]
Li et al.	2017	China	RS	N/A	U	29+31	2	CCRT (AP=PC)	59.6	[27]

Abbreviations: AP, pemetrexed plus cisplatin; CCRT, concurrent chemoradiotherapy; CF, carboplatin plus 5‐fluorouracil; DCF, docetaxel, cisplatin, 5‐fluorouracil; dCRT, definitive chemoradiotherapy; DP, docetaxel plus cisplatin; ECX, epirubicin plus cisplatin plus capecitabine; ICT, induction chemotherapy; nCRT, neoadjuvant chemoradiotherapy; PC, paclitaxel plus carboplatin; PF, cisplatin plus 5‐fluorouracil; PS, prospective; P‐S1, cisplatin plus TS‐1; R, resectable; RS, retrospective; RT, radiotherapy; SCRT, sequential chemoradiotherapy; U, unresectable.

In our study, PC showed a high survival benefit, consistent with the findings of the CROSS study ^12^ and other Asian studies.^18^, ^20^ Other regimens using non‐taxane agents, different schedules, or different intensities of radiotherapy have also been explored.^22^, ^23^, ^24^, ^25^, ^26^, ^27^ These reported regimens included the SOX (oxaliplatin‐TS‐1) regimen,^22^ FOLFOX regimen,^28^ ECX (epirubicin, cisplatin, and capecitabine),^29^ pemetrexed plus cisplatin,^27^ neoadjuvant CRT versus chemotherapy with PF regimen before surgery,^23^ concurrent CRT and sequential CRT with capecitabine and cisplatin in elderly patients,^24^, ^26^ a higher dose or RT (>60 Gy) with the PF regimen,^25^ and RT alone in the elderly.^26^ However, there is still no evidence to support changing the current practice guidelines to concurrent CRT. The success of immunotherapy as the second‐line therapy^30^, ^31^ in patients with recurrent or metastatic EC might someday contribute to the development of new, more combination regimens with radiotherapy.

### 4.2 Impact of complete pathological remission

A study by Haisley et al. (2017) reported that the PF regimen is better than the PC regimen with respect to recurrence‐free survival and the OS benefit which could be due to a higher pathological complete remission rate (pCR) (33% vs. 22%).^11^ However, pCR was not analyzed in their multivariate regression model, which may have resulted in better survival outcomes in the PF group that had a higher pCR rate. In contrast, the pCR rates in the current study were 7.4% (*n* = 4/54) and 10% (3/30) in the PF and PC groups, respectively (*p* = 0.755), resulting in an overall pCR rate of 8.3% (7/84). The relatively low pCR rate in the present study was due mainly to (i) the population (neoadjuvant vs. definitive CCRT) and (ii) the post‐concurrent CRT surgical rate of 100.0% vs. 36.7% (84/229) in our study and Haisley et al.’s report, respectively. Collectively, these results support the line of thinking that PC may be better than PF during dCRT for patients with advanced EC.

### 4.3 Limitations of the study

There were some limitations in the current study. First, its retrospective nature limited the power of the scientific conclusion. However, except for the neoadjuvant PC regimen from the CROSS study in 2012, to the best of our knowledge, no prospective phase III study evaluating the regimens in concurrent CRT has been reported. Further, our study involved a relatively large population of inoperable patients with EC who received definitive CRT. Second, owing to the chart‐review design, the data on several toxicity profiles were incomplete, and thus, we could not evaluate the differences in toxicity between the PF and PC regimens. Third, we only compared the PC and PF regimens and did not enroll patients who received other regimens. Our findings need to be validated in prospective randomized phase III trials to establish the optimal chemotherapy regimen during dCRT for EC. Fourth, the patients did not choose their regimens randomly as is done in prospective trials. Thus, the final analysis may have been influenced by a selection bias. However, our comparison of the two groups in Table 1 indicated no apparent differences in the two groups’ essential characteristics.

In conclusion, PC yielded superior OS and PFS benefits compared to PF as the chemotherapy regimen during dCRT for patients with advanced ESCC. On the basis of the findings from real‐world evidence, we propose that the PC regimen may be a preferable chemotherapy choice for dCRT in patients with advanced inoperable EC.

## CONFLICT OF INTERESTS

The authors declare that they have no competing interests.

## Data Availability

All data generated or analyzed during this study are included in this published article.
